# Beliefs of US chiefs of police about substance use disorder, fentanyl exposure, overdose response, and use of discretion: results from a national survey

**DOI:** 10.1186/s40352-025-00318-8

**Published:** 2025-03-05

**Authors:** Amelia Bailey, Barbara Andraka-Christou, Saba Rouhani, M.H. Clark, Danielle Atkins, Brandon del Pozo

**Affiliations:** 1 Brown University, Providence, USA; 2 University of Central Florida, Orlando, USA; 3 New York University, New York, USA; 4 Florida State University, Tallahassee, USA; 5 Rhode Island Hospital, Providence, USA

**Keywords:** Police, Addiction, Diversion, Harm reduction, Fentanyl exposure, Discretion, Survey, Buprenorphine, Marijuana

## Abstract

**Background:**

To inform the feasibility and acceptability of evidence-informed police practices related to substance use, addiction, and overdose, we sought to better understand how US police chiefs perceive substance use and related policing practices.

**Methods:**

A national sample of randomly selected US police chiefs (*N* = 276) completed a 37-item survey about substance use and policing. Nine items assessed chiefs’ perceptions of: officers’ discretion in making arrests, effectiveness of overdose responses, risks of fentanyl exposure, de-escalation practices, harmful drugs in their community, and illicitly-obtained buprenorphine. Data were analyzed with descriptive statistics and exploratory ordinal logistic regressions.

**Results:**

Most chiefs (72.5%) agreed that arrest for any nonviolent misdemeanor was at the discretion of their officers, and they overwhelmingly (94.9%) trusted their officers to make the right arrest decision. The majority of chiefs (87.7%) felt their officers could effectively respond to an opioid overdose, and 83.7% reported their officers carried naloxone on patrol. Chiefs in the Northeast were significantly less likely to be confident in their officers’ ability to respond to a methamphetamine overdose than chiefs in the West. Most (90.0%) were receptive to implementing methamphetamine de-escalation strategies (i.e., techniques to resolve crises short of force). Almost all chiefs (91.2%) agreed with the inaccurate statement that fentanyl exposure at a drug overdose scene could harm officers.

**Conclusions:**

Police chiefs express interest in several types of evidence-based public health approaches to policing. Critically, there is a need to curtail fentanyl misinformation and to improve officer knowledge about medications for treating opioid use disorder.

## Introduction

Over 85% of all people who use drugs (PWUD) report a lifetime history of arrest, with one-third reporting a recent encounter with law enforcement (Degenhardt et al., 2017). Such police practices can have significant effects on the health of this population (Grella et al., 2020; Mackey et al., 2020; National Academies of Sciences & Medicine, 2019; Sugarman et al., 2020). Given evidence that incarceration can increase exposure to risk factors for and incidence of overdose (Keen et al., 2020; O’Connor et al., 2022; Pizzicato et al., 2018), alternatives to arrest for drug possession should be explored. For example, police officers in some municipalities can connect PWUD to treatment and harm reduction resources (del Pozo et al., 2021a; Pozo et al., 2021b). However, more information is needed to understand police beliefs about practices informed by public health and harm reduction goals, as beliefs may predict behavior and impact the feasibility of such interventions (Lloyd et al., 2023; Wurcel et al., 2023). For example, even if police have discretion to not arrest PWUD for drug possession, police might worry that discretion would be used inequitably or could worsen public safety outcomes (Feys, 2023; Knode et al., 2024; Samuels-Wortley, 2022).

Police also often respond to overdose events (Carroll et al., 2020; Pike et al., 2021; Ray et al., 2015), but the extent to which they ought to do so remains unclear, since people may hesitate to seek help for an overdose victim out of fear of arrest (Atkins et al., 2024; del Pozo, 2022; Rouhani et al., 2021), which has been shown to occur as often as 10% of the time (Ray et al., 2022). Studies have also revealed deeply misinformed fears among police about the dangers of accidental exposure to fentanyl (Attaway et al., 2021; del Pozo et al., 2022; del Pozo, Sightes, Pozo et al., 2021a, b; Siegel, 2022). Such fears may prevent officers from rapidly engaging with overdose victims when time is of the essence.

Chiefs of police are the municipal executives who devise the strategies and policies that affect police officers’ behavior; and chiefs set the expectations for mid-level supervisors, who have considerable influence over the behavior of the officers who routinely interact with community residents (i.e., “rank-and-file” officers) (del Pozo et al., 2021a; Pozo et al., 2021b; Marotta et al., 2023; Wurcel et al., 2023). Therefore, an understanding of chiefs’ beliefs and perceptions is critical for contextualizing the nation’s municipal responses to substance use and overdose, determining what police-related interventions are likely to be feasible and acceptable, and identifying where gaps in knowledge among police may prevent the most effective responses.

To help provide an understanding of these beliefs and perceptions, we conducted a randomized national survey of US chiefs of police. We assessed their use of and interest in harm reduction practices, as well as their beliefs about substances in the community.

## Materials and methods

### Ethics

This study was designed and conducted in accordance with the principles of the Common Rule. It was determined not to exceed minimal risk by Rhode Island Hospital’s Institutional Review Board, which waived written consent and declared it exempt. All survey respondents received an Explanation of Research prior to data collection.

### Participants and study design

This analysis utilized data from a larger national survey of US municipal chiefs of police, and the methods presented here correspond to those described in published analyses of other data from the instrument (del Pozo et al. (2024)). To capture the views of police executives operating in more complex organizational and political environments, our sample was derived from the population of 9,673 municipal police departments in the US that a commercial police data vendor reported as employing five or more sworn officers in 2022 (NPSIB, 2022). From this group, 1,200 randomly selected chiefs were invited to participate in the survey. To assess respondents’ sensitivity to the framing of items in ways that broadly emphasized a public health vernacular vs. one that used public safety terminology, two versions of four of this study’s items were administered to chiefs: *n* = 600 were randomly assigned to the “health” framed items, and the remaining *n* = 600 were assigned to “safety” framed items. In short, each version of the survey measured the same constructs with mostly identical phrasing, but one set of questions used language about preventing health harms, while the other used language typically found in discussions of public safety and law enforcement (Table 1). The survey was mailed to chiefs in late 2022 and early 2023 in two waves, the second of which was limited to the nonrespondents of the first.

**Table 1:** Survey items relevant to police chief knowledge, attitudes, and beliefs on addiction and policing

Items		
**Demographic items**		**Unit**
1. How many years have you served in policing?		Years
2. How many years have you served as an agency head?		Years
3. Approximately how many full-time sworn officers do you lead?		Number of officers
**Substantive items**		**Likert scale anchors** ^**c**^
1. My police officers know how to effectively respond to opioid overdoses.		Strongly disagree/ Strongly agree
2. My police officers know how to effectively respond to methamphetamine overdoses.		Strongly disagree/ Strongly agree
3. If there was a way to de-escalate people high on methamphetamines and get them to medical treatment, I’d implement it. ^b^		Strongly disagree/ Strongly agree
4. Police should be cautious in responding to drug overdose scenes because fentanyl may be present, and it can harm them. ^b^		Strongly disagree/ Strongly agree
5. Do the police officers in your agency carry Narcan (naloxone) on patrol?		Yes/ No
*Health-related frame*	*Safety-related frame*	
6. Whether police officers in my agency arrest a *person* for any nonviolent misdemeanor is:	Whether police officers in my agency arrest a *drug user* for any nonviolent misdemeanor is:	Not at their discretion at all/ Entirely at their discretion
7. I trust my officers to make the right decision about whether to arrest a *person* for a nonviolent misdemeanor.	I trust my officers to make the right decision about whether to arrest a *drug user* for a nonviolent misdemeanor.	Strongly disagree/ Strongly agree
8. People illegally use buprenorphine (Suboxone) to *manage their opioid addiction on their own*. ^b^	People illegally use buprenorphine (Suboxone) to *get high with a less dangerous drug than heroin or fentanyl*. ^b^	Strongly disagree/ Strongly agree
9. Please rank the following substances from 1 to 6 in terms of their relative *harms to* the community you serve: ^a^	Please rank the following substances from 1 to 6 in terms of their relative *threat to the safety of* the community you serve: ^a^	Most harmful/ Least harmful

*a = Cocaine; Heroin/fentanyl/opioids; Alcohol; Methamphetamine; Cannabis; Crack cocaine*

*b = Participants could respond “don’t know”*

*c = Responses were ranked from 1 to 7 (lower anchor to upper anchor)*

### Measures

Of the complete survey’s 37 items, the present study considered nine items related to chiefs’ perceptions and beliefs about harm reduction and public health-informed practices. Specifically, the items assessed the following: perceived harms of accidental exposure to fentanyl; officers’ use of discretion in making drug-related arrests; trust in an officer’s ultimate decision to arrest; perceived relative hazards of various substances; officer carriage of naloxone on patrol; effective responses to opioid and methamphetamine overdoses; interest in methamphetamine incident de-escalation techniques; a chief’s trust of officers in recovery for opioid dependency; and use of illicitly-possessed buprenorphine (see Table 1 for the list of items). Responses were measured using a semantic differential scale (i.e., 1 to 7) with the appropriate anchoring language, or a “yes” or “no” response as appropriate. Respondents also completed items about individual and agency demographics.

### Data analysis

Responses to the survey items were transformed prior to our analyses. To more clearly describe the data collected, six of the ordinal items with responses ranging from 1 to 7 were categorized as strong disagreement (1), any disagreement (1–3), neutral (4), any agreement (5–7), and strong agreement (7). For the remaining two ordinal items, one had a range from “no discretion” to “complete discretion,” and was transformed according to the numerical ordinal responses; and the other had a range from “least harmful” to “most harmful,” where respondents were asked to rank each substance relative to the others. Whether an agency’s officers carry naloxone on patrol was presented as a yes or no question and coded as a 1 or 0. These transformed data were used to derive our results.

Data were analyzed in two steps. First, descriptive statistics were used to describe trends by responses (mean and standard deviation; frequency and proportion). Upon examination of the responses, descriptive differences were observed for some items by agency characteristics. To better explore these differences, adjusted ordinal logistic regressions inclusive of all predictor variables were used to explore how agency characteristics (i.e., US Census Bureau region, urbanicity, length of time in the department before becoming chief, length of time as chief, and ratio of agency to population size) were associated with response data. These regressions utilized the original continuous data provided by respondents (i.e., ranging from 1 to 7). Analyses were conducted in Stata v18 (StataCorp, 2023).

## Results

The final sample consisted of 276 respondents, for a response rate of 23%. The mean number of years responding chiefs had served in policing was 28.4 (SD = 8.5), with an average of 6.9 years (SD = 6.6) as a chief. Respondents’ agency size varied from 5 to 460, with a median of 15. Most of the sample worked in the Midwest Census Bureau Region (39.9%), followed by the South (27.9%) and Northeast (21.0%), with the fewest participants from the West (11.2%), serving 4.6 million US residents. The communities served were most often micro- or metropolitan (77.5%) rather than small or rural towns (22.5%). The largest responding agencies had up to 500 officers and served up to 300,000 residents; responding chiefs collectively served a population of 4.6 million. There were no significant differences observed between respondents and nonrespondents except that agencies from the Southern Census Bureau Region were significantly less likely to respond to the survey than chiefs from the Midwest (18.9% vs. 29.4%). Respondent demographics and analyses of response and nonresponse are presented at length in del Pozo et al. (2024). Using Wilcoxon rank sum tests, there were no significant differences by item in the responses to each arm of our sensitivity analyses, so the results below are presented in the aggregate.

### Use of discretion

Regardless of whether the subject was characterized as a “person” or a “drug user,” most chiefs (72.5%) agreed that arrest for a nonviolent misdemeanor was at the discretion of their officers, and the vast majority of chiefs (94.9%) trusted their police officers to make the “right” decision about the use of discretion (Table 2).

**Table 2:** Police chief responses (*n* = 276) to addiction and policing items

Item (lower anchor/upper anchor)	Item *n* (%)	Response *n* (%), or Health *n* (%)-Safety *n* (%)							
		1		1–3		4		5–7	7
1. **Officer discretion in arrest (not at discretion/at discretion)**^**a**^	276 (100%)	3 (2.1%)-5 (3.8%)		14 (9.8%)-18 (13.5%)		21 (14.7%)-23 (17.3%)		108 (75.5%)-92 (69.2%)	32 (22.4%)-34 (25.6%)
2. **Officer trust in arrest (strongly disagree/strongly agree)**^**b**^	275 (99.6%)	1 (0.7%)-1 (0.8%)		3 (2.1%)-3 (2.3%)		7 (4.9%)-1 (0.7%)		132 (93.0%)-129 (97.0%)	57 (40.1%)-70 (52.6%)
3. **Effective opioid overdose response (strongly disagree/strongly agree)**	276 (100%)	0 (0.0%)		12 (4.3%)		22 (8.0%)		242 (87.7%)	77 (27.9%)
4. **Effective methamphetamine overdose response (strongly disagree/strongly agree)**	276 (100%)	3 (1.1%)		28 (10.1%)		46 (16.7%)		202 (73.2%)	46 (16.7%)
5. **Interest in methamphetamine de-escalation (strongly disagree/strongly agree)**	260 (94.2%)	3 (1.2%)		7 (2.7%)		19 (7.3%)		234 (90.0%)	97 (37.3%)
6. **Officer risk of fentanyl exposure (strongly disagree/strongly agree)**	273 (98.9%)	7 (2.6%)		12 (4.4%)		12 (4.4%)		249 (91.2%)	152 (55.7%)
7. **Reason for illegal use of buprenorphine (strongly disagree/strongly agree)**^**c**^	154 (55.6%)	3 (3.3%)-6 (9.4%)		11 (12.2%)-9 (14.1%)		24 (26.7%)-14 (21.9%)		55 (61.1%)-41 (64.1%)	12 (13.3%)-11 (17.2%)
		**1st**	**2nd**		**3rd**		**4th**	**5th**	**6th**
8. **Rank substance (most harmful/least harmful)**^**d, e**^									
Cocaine	272 (98.6%)	1 (0.7%)-3 (2.3%)	6 (4.3%)-14 (10.6%)		25 (17.9%)-22 (16.7%)		43 (30.7%)-47 (35.6%)	51 (36.4%)-38 (28.8%)	14 (10.0%)-8 (6.1%)
Heroin/fentanyl/opioids	272 (98.6%)	80 (57.1%)-69 (52.3%)	29 (20.7%)-33 (25.0%)		14 (10.0%)-17 (12.9%)		12 (8.6%)-9 (6.8%)	1 (1.4%)-2 (1.5%)	3 (2.1%)-2 (1.5%)
Alcohol	271 (98.2%)	26 (18.6%)-27 (20.8%)	30 (21.4%)-16 (12.2%)		36 (25.7%)-29 (22.0%)		14 (10.0%)-15 (11.5%)	20 (14.3%)-19 (14.5%)	14 (10.0%)-24 (18.5%)
Methamphetamine	271 (98.2%)	28 (20.0%)-33 (25.2%)	56 (40.0%)-51 (38.9%)		28 (20.0%)-20 (15.3%)		13 (9.3%)-10 (7.6%)	8 (5.7%)-8 (6.1%)	7 (5.0%)-9 (6.9%)
Cannabis	272 (98.6%)	5 (3.4%)-3 (2.3%)	10 (7.1%)-9 (6.8%)		14 (10.0%)-13 (9.9%)		23 (16.4%)-23 (17.4%)	16 (11.4%)-29 (22.0%)	72 (51.4%)-55 (41.7%)
Crack cocaine	272 (98.6%)	1 (0.7%)-2 (1.5%)	11 (7.9%)-9 (6.8%)		21 (15.0%)-32 (24.2%)		36 (25.7%)-30 (22.7%)	39 (27.9%)-25 (18.9%)	32 (22.9%)-34 (25.8%)

* denotes significance at P < *0.01*

** denotes significance at P < *0.05*

^a^ One arm referred to the arrested individual as a “person” and the other as a “drug user”

^b^ One arm referred to the arrested individual as a “person” and the other as a “drug user”

^c^ One arm referred to illicit use as intending “to manage their opioid addiction on their own“ and the other as intending “to get high with a less dangerous drug than heroin or fentanyl“

^d^ One arm asked participants to rank the substance in its “relative harms to the community you serve” and the other in its “relative threat to the safety of the community you serve”

^e^ There were no statistically significant differences (*p* < 0.05) by health or safety frame for the framed questions

### Overdose response

Of the 276 respondents, 83.7% reported that their officers carry naloxone on patrol. A great majority of chiefs (87.7%) believed their officers could effectively respond to an opioid overdose. Considerably fewer believed their officers could effectively respond to a methamphetamine overdose (73.2%), with 10.1% disagreeing their officers could do so. Almost all chiefs (90.0%) agreed that they would implement an effective way to “de-escalate people high on methamphetamines and get them to medical treatment.”

### Fentanyl exposure misinformation

Almost all chiefs (91.2%) agreed with the inaccurate statement that accidental exposure to fentanyl at an overdose scene was a significant threat to their officers, therefore requiring caution, with more than half (55.7%) strongly agreeing.

### Most harmful substances in the community

More than half of chiefs (54.8%) identified opioids (e.g., heroin, fentanyl, opioid analgesics) as the most harmful substance in their community as compared to other substances. Methamphetamine was the second substance most commonly perceived as most harmful (22.5% of respondents). Marijuana was consistently cited as the least harmful substance of the ones presented, and the others filled the middle ranks. Opioids were perceived as less harmful by chiefs from small or rural towns as compared to those from micro- or metropolitan areas (*p* = 0.033). Chiefs in all other regions of the US were more likely to agree that methamphetamine was the most harmful substance in their community than chiefs in the Northeast (South: *p* < 0.001; Midwest: *p* < 0.001; West: *p* = 0.001); chiefs in small or rural towns were more likely to agree that methamphetamine was most harmful to their community compared to micro- or metropolitan areas (*p* = 0.022). See Table 3.

**Table 3:** Exploratory regression analyses examining associations between responses and demographic/agency characteristics

	Odds ratios and 95% confidence intervals									
	Discretion ^a^	Trust ^b^	Opioid response	Methamphetamine response	Methamphetamine de- escalation	Fentanyl risk	Cocaine less harmful ^d^	Opioids less harmful ^d^	Methamphetamine less harmful ^d^	Crack cocaine less harmful ^d^
**Census regions**										
Northeast	Reference									
South	1.50 (0.80, 2.82)	1.17 (0.59, 2.31)	0.36 (0.19, 0.67) **	1.64 (0.85, 3.17)	0.91 (0.47, 1.78)	0.89 (0.44, 1.80)	0.99 (0.47, 1.70)	1.58 (0.76, 3.28)	0.30 (0.15, 0.58) **	0.82 (0.43, 1.54)
Midwest	1.09 (0.60, 1.97)	1.20 (0.64, 2.24)	0.56 (0.31, 1.02) *	2.11 (1.15, 3.90) **	1.57 (0.85, 2.91)	0.82 (0.43, 1.55)	0.99 (0.56, 1.78)	1.76 (0.91, 3.39) *	0.33 (0.18, 0.61) **	1.67 (0.92, 3.02) *
West	1.66 (0.75, 3.68)	1.65 (0.70, 3.89)	0.90 (0.41, 1.99)	4.38 (1.96, 9.80) **	1.97 (0.83, 4.68)	0.67 (0.30, 1.53)	1.67 (0.75, 3.72)	1.26 (0.53, 2.99)	0.24 (0.10, 0.53) **	2.07 (0.95, 4.55)
**Urbanicity**										
Metro/micropolitan	Reference									
Small town/rural	1.07 (0.63, 1.80)	0.73 (0.41, 1.28)	0.53 (0.31, 0.90) **	1.03 (0.62, 1.70)	0.47 (0.27, 0.80) **	1.44 (0.80, 2.60)	1.66 (0.97, 2.83)	1.80 (1.03, 3.16) **	0.53 (0.31, 0.91) **	1.09 (0.64, 1.84)
**Tenure**										
Years as chief	1.02 (0.99, 1.06)	1.00 (0.97, 1.04)	1.01 (0.98, 1.05)	1.02 (0.99, 1.06)	1.01 (0.97, 1.04)	0.99 (0.95, 1.02)	0.96 (0.93, 0.10) **	1.03 (0.99, 1.07)	0.96 (0.93, 0.99) **	0.99 (0.95, 1.02)
Years not as chief	1.01 (0.98, 1.04)	1.01 (0.98, 1.04)	0.99 (0.97, 1.03)	1.02 (0.99, 1.05)	1.01 (0.98, 1.05)	0.10 (0.97, 1.03)	0.98 (0.96, 1.01)	0.99 (0.96, 1.02)	1.03 (0.99, 1.06) *	0.98 (0.95, 1.00)
**Staffing**										
Residents per officer	0.95 (0.85, 1.05)	1.03 (0.92, 1.15)	1.02 (0.92, 1.14)	1.10 (0.98, 1.22) *	1.01 (0.91, 1.13)	0.92 (0.82, 1.04)	1.03 (0.92, 1.15)	1.06 (0.94, 1.19)	0.90 (0.80, 0.99) **	1.05 (0.94, 1.18)

* denotes significance at P < *0.01*

** denotes significance at P < *0.05*

^a^ One arm referred to the arrested individual as a “person” and the other as a “drug user”

^b^ One arm referred to the arrested individual as a “person” and the other as a “drug user”

^c^ One arm referred to illicit use as intending “to manage their opioid addiction on their own“ and the other as intending “to get high with a less dangerous drug than heroin or fentanyl“

^d^ One arm asked participants to rank the substance in its “relative harms to the community you serve” and the other in its “relative threat to the safety of the community you serve”

### Use of non-prescribed buprenorphine

Half of the survey recipients were asked the extent to which PWUD use illicit buprenorphine to manage their opioid addiction, while the other half were asked the extent PWUD used illicit buprenorphine to get high with a less dangerous opioid than other opioids. Across both items, 43.1% (*n* = 119) of respondents answered “I don’t know,” while nearly one-third of participating chiefs chose to not respond to the item at all. Of those who responded on the semantic differential scale, 24.7% (*n* = 38) neither agreed nor disagreed with the proposition they were evaluating (Table 2).

Chiefs’ perceptions of whether their officers could effectively respond to an overdose varied by urbanity and geographic location (Table 3). Chiefs from small or rural towns were half as likely to agree than police from micro- or metropolitan areas that officers could effectively respond to opioid overdoses (*p* = 0.019). Chiefs in the Midwest and West were more likely than those in the Northeast to agree that their officers could effectively respond to a methamphetamine overdose (*p* = 0.016; *p* < 0.001). Chiefs from small and rural towns expressed less interest in de-escalation strategies for a person intoxicated by methamphetamine (*p* = 0.006).

## Discussion

To our knowledge, this is the first national survey of US chiefs of police about substance-use issues of relevance to present public health and safety concerns. Our findings suggest most chiefs believe their officers have discretion in arresting people for nonviolent misdemeanors, such as the ones related to problematic substance use, and trust their officers to use discretion properly, a belief shared by officers themselves in another study (del Pozo et al., 2021). Our findings reinforce the potential feasibility and acceptability among police of discretionary diversion to substance use treatment as an alternative to arrest in a range of cases. To support discretionary linkages to treatment and harm reduction, chiefs would need to provide clear and persuasive policy directives to mid-level supervisors who oversee and directly influence the behavior of the rank-and-file officers most likely to interact with PWUD (del Pozo et al., 2021b; Marotta et al., 2023; Reichert et al., 2023).

We found notable regional variation in perceptions about the most harmful substances affecting a community. As compared to police chiefs in the Northeast, police chiefs in the West were more likely to agree that methamphetamine was the most harmful. These beliefs and attitudes are consistent with historical trends in the drug market and substance use patterns across the US, where methamphetamine has been prominent in the West Coast drug supply for considerably longer than in the Northeast (Ciccarone, 2021). Importantly, relative to other substances, officers were least likely to consider marijuana the most harmful substance in their community – a finding that is particularly relevant as the Federal Drug Enforcement Administration is evaluating whether to reschedule marijuana from Schedule I (most restrictive) to Schedule III of the Controlled Substances Act (DEA, 2024). Currently, both heroin (an opioid) and marijuana are within the same schedule, yet officers clearly do not believe they are equally harmful to their communities – even despite the increasingly widespread use of recreational and medical marijuana in US states (Hasin & Walsh, 2021).

The extensive carriage of naloxone by patrol officers observed in this study indicates overdose response is perceived by US municipal police chiefs to be one of their officers’ core roles. Interestingly, chiefs in the Northeast were significantly less likely to express confidence in their officers’ ability to respond to stimulant overdoses, possibly owing to less experience in doing so, as methamphetamine has historically been more commonly used by PWUD in the West than the Northeast (Mattson et al., 2021). This deficit will presumably become more acute, as stimulant-related overdose deaths rose 43.8% in the Northeast from 2018 to 2019, vs. 17.5% in the West (Mattson et al., 2021), reflecting rising burdens on emergency response and hospital services in the Northeast (Bettano et al., 2022; Calcaterra et al., 2024). Therefore, chiefs will need to implement policies that assist police officers in addressing stimulant overdoses.

Chiefs’ widespread willingness to adopt a de-escalation curriculum tailored for stimulants that links patients to effective medical care highlights the need for such research and the potential acceptability of the results. Promisingly, our findings suggest chiefs are largely interested in de-escalation strategies; but chiefs may need to first address negative attitudes among intermediary officers and rank-and-file officers about de-escalation. Prior research has found that police officers often perceive methamphetamine incidents as more likely to result in violence (Jones et al., 2022) and have associated methamphetamines with the controversial diagnosis of excited delirium to justify police use of force (Obasogie, 2021).

Unfounded risks associated with officer exposure to fentanyl have been sensationalized in the media through the spread of misinformation (ABC News, 2021; Attaway et al., 2021; del Pozo et al., 2022; del Pozo, Sightes, Pozo et al., 2021a, b; Morris, 2022; Siegel, 2022). Chiefs in the present study overwhelmingly agreed with the inaccurate statement that accidental fentanyl exposure posed a threat to officers. Since chiefs are influential within their agencies, such beliefs may further inhibit officers’ willingness to respond to overdoses and exercise an appropriate response (del Pozo et al., 2022). Unfounded fears about accidental exposure to fentanyl could also prompt police agencies to inefficiently use funds, such as by buying unnecessary personal protective equipment for use in responding to overdose (Centers for Disease Control and Prevention, 2019).

While respondents were equally likely to agree that non-prescribed buprenorphine was used to manage addiction as they were to agree that it was used to “get high”, over 40% of respondents did not know why a person might use the medication illicitly, and one-third of the sample did not respond to this item at all, making it by far the most unanswered item in the survey. This suggests a lack of understanding among police chiefs about the properties of buprenorphine, and how it is used to treat opioid use disorder. Buprenorphine has been shown to significantly reduce opioid overdose (Wakeman et al., 2020) including when used illicitly as a form of self-treatment for substance use disorder (Fox et al., 2015; Schuman-Olivier et al., 2010). Given it poses very little risk of overdose itself (del Pozo et al., 2023) and some jurisdictions have decriminalized it when possessed without a prescription (del Pozo et al., 2020), US police leaders would benefit from such an understanding of how buprenorphine works.

### Limitations

As discussed in del Pozo et al. (2024), this study has limitations. First, we grouped 7-point Likert-scale responses into differing levels of agreement to describe our results in a clearer and more intuitive way, but this reduced the ability of our analysis to detect more subtle variability in the underlying data. Second, the study sample is comprised of chiefs from police departments with five or more officers, which excluded the perspective of chiefs from smaller locales. Third, the overall response rate to this survey was 23%, on the low end for generalizable survey data collected by mail, but this rate is typical of surveys of police (e.g., (Korre et al., 2014; Nix et al., 2019; PERF, 2023; Telep, 2016), and exceeds most published survey research studying US chiefs of police, which are a hard-to-reach research population. While the ability to generalize from the study’s data is enhanced by its random sampling approach, results that compare Census Bureau regions should be interpreted with caution given the small subsamples that result from dividing up responses in this manner. Additionally, while a mean respondent tenure of 28.4 years exceeds the average 26.4-year length of a police career at the time of full retirement observed in a notable study (Raub, 1987), respondents had ascended to the rank of chief, which suggests a longer investment in policing than lower-ranking officers. Finally, since the sampling frame was selected at random from the entire population of eligible chiefs, and there were no significant differences observed between respondents and nonrespondents with the exception of Census Bureau Region, there is no cause to believe the sample analyzed here is unique in terms of tenure, or in other demographic regards.

## Conclusion

This randomized national survey study of municipal chiefs of police, who collectively serve more than 4.6 million residents, found that most granted their officers considerable discretion in making arrests for the nonviolent misdemeanors typically associated with problematic substance use. Therefore, diversion to treatment as an alternative to arrest may be feasible and acceptable to many police leaders. Chiefs in our study generally felt their officers effectively respond to opioid overdoses but felt less confident with respect to stimulant overdoses. Regional variation in responses mirrored previous reports of the “fourth wave” of the opioid crisis. Fortunately, the majority of chiefs indicated they would be interested in de-escalation strategies for responding to incidents involving stimulant use – an important topic given the increasing use of stimulants nationally, including in combination with opioids. Future interventions with chiefs should aim to correct rampant misinformation about the dangers of fentanyl exposure in the field and enhance police executives’ knowledge about the properties and uses of medications for opioid use disorder, especially buprenorphine.

## Data Availability

The datasets generated and/or analyzed during the current study are available from the corresponding author upon reasonable request.

## Abbreviations

PWUD: People who use drugs
US: United States
